# Coexisting Non-inflammatory Fibrotic Entrapment and Traumatic Neuroma as Distinct Mechanisms of Anterior Cutaneous Nerve Entrapment Syndrome (ACNES) Following Laparoscopic Percutaneous Extraperitoneal Closure (LPEC) in a Child

**DOI:** 10.7759/cureus.108070

**Published:** 2026-04-30

**Authors:** Kyeong Deok Lee, Yuki Nagasako

**Affiliations:** 1 Pediatric Surgery, TMG Asaka Medical Center, Saitama, JPN; 2 General Medicine, TMG Asaka Medical Center, Saitama, JPN; 3 Pediatric Surgery, Juntendo University Nerima Hospital, Tokyo, JPN; 4 Pediatric Surgery, Juntendo University School of Medicine, Tokyo, JPN

**Keywords:** acnes, anterior cutaneous nerve entrapment syndrome, chronic post-surgical pain, laparoscopic inguinal hernia repair, lpec, traumatic neuroma

## Abstract

Anterior cutaneous nerve entrapment syndrome (ACNES) is an important cause of focal abdominal wall pain and may be missed when routine imaging and laboratory findings are normal. We report an eight-year-old girl who developed sharp, well-localized right lower abdominal pain six months after bilateral laparoscopic percutaneous extraperitoneal closure (LPEC) for inguinal hernia. She was initially evaluated at a university hospital and diagnosed with irritable bowel syndrome; however, the pain progressed and prevented school attendance. On presentation to our department, she had a clear focal tender point to the right of the umbilicus with a positive Carnett sign, consistent with ACNES. One day before surgery, she additionally reported new localized pain at a pigmented lesion in the right groin, and a neurectomy was performed at two sites. At the periumbilical site, a cutaneous nerve was identified immediately beneath the skin incision and excised with the fascia. Pathology demonstrated fibrosis around the neurovascular bundle, thickened nerve fascicles, and close apposition of the nerve fascicles to an artery. At the right groin site, no cutaneous nerve was found directly beneath the skin; a cutaneous nerve branch extending cranially was identified on deeper exploration and excised with the fascia. Pathology demonstrated adipose scar formation and a perineural/fascial neuroma with whorled nerve fascicles intertwined with collagen fibers. Postoperatively, pain at both sites resolved promptly without recurrence. This case illustrates that distinct mechanisms of abdominal wall neuropathic pain, including ACNES and traumatic neuroma, may coexist after LPEC.

## Introduction

Chronic postoperative pain is a common and clinically relevant problem, affecting approximately 2-10% of patients after various surgical procedures, and represents a major cause of long-term morbidity [1,2]. Although minimally invasive techniques have reduced tissue trauma and early postoperative discomfort, neuropathic pain syndromes remain an important and often underrecognized complication of surgery. In particular, postoperative abdominal wall pain may persist despite normal laboratory findings and imaging studies, leading to delayed diagnosis and prolonged functional impairment [3].

Anterior cutaneous nerve entrapment syndrome (ACNES) is a well-described cause of focal abdominal wall pain resulting from the entrapment of terminal branches of the intercostal nerves as they traverse the abdominal musculature [3-5]. Clinically, ACNES is characterized by a sharply localized tender point, often accompanied by a positive Carnett sign, and may mimic visceral or functional gastrointestinal disorders [3]. Consequently, affected patients are frequently misdiagnosed with conditions such as irritable bowel syndrome, especially when routine investigations are unrevealing [3]. Histopathological features of ACNES have not been fully characterized, and reported findings range from minimal structural changes to fibrosis and nerve thickening, suggesting heterogeneous underlying mechanisms [6].

Laparoscopic percutaneous extraperitoneal closure (LPEC) is widely used for inguinal hernia repair, particularly in pediatric patients, because of its minimal invasiveness and favorable cosmetic outcomes [7]. However, trocar placement, needle puncture, and fascial manipulation during LPEC may place adjacent cutaneous nerve branches at risk of injury or entrapment. In addition to classic nerve entrapment, traumatic neuroma formation along puncture or suture tracts represents another potential source of postoperative neuropathic pain, further complicating clinical differentiation [8].

While ACNES and traumatic neuroma are typically discussed as separate entities, their coexistence in a single patient has rarely been described [8]. Understanding the distinct yet potentially overlapping mechanisms of postoperative abdominal wall neuropathic pain is essential for accurate diagnosis and lesion-directed management [1,2,8]. Here, we report a pediatric case in which non-inflammatory fibrotic entrapment of a cutaneous nerve and traumatic neuroma formation appeared to coexist after bilateral LPEC, with distinct intraoperative and histopathological findings suggestive of different underlying pain generators.

## Case presentation

An eight-year-old girl with a history of asthma underwent bilateral LPEC for an inguinal hernia at another hospital and initially had an uncomplicated postoperative course. Six months later, she developed sharp, localized pain in the right lower abdomen without an apparent trigger. She was evaluated at a university hospital, where investigations were unrevealing and she was diagnosed with irritable bowel syndrome; however, her symptoms persisted. Because the pain became severe enough to prevent school attendance, her primary physician suspected an abdominal wall pain source and referred her to our department for possible ACNES.

**Figure 1:**
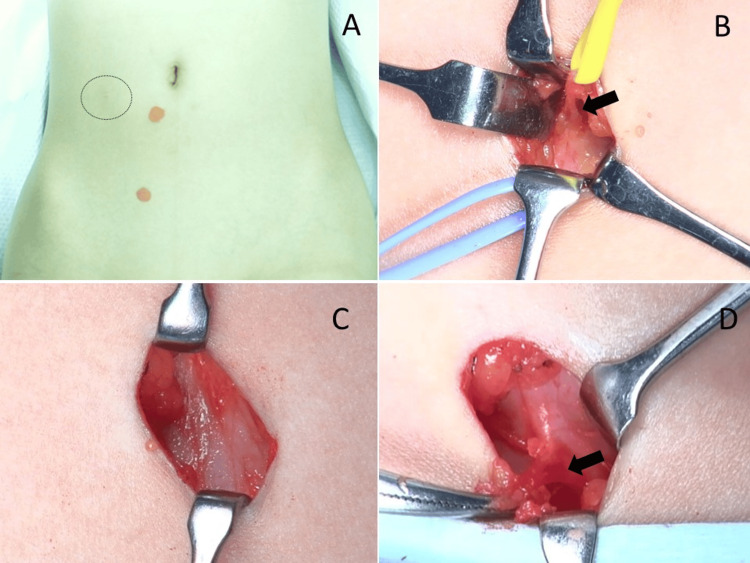
Operative findings of postoperative abdominal wall pain lesions (A) Preoperative photograph of the periumbilical region. The red skin mark indicates the site of maximal tenderness, while the dotted circle denotes the prior trocar insertion site from bilateral laparoscopic percutaneous extraperitoneal closure. The presumed needle puncture site was not clearly identifiable on the skin surface. (B) Intraoperative view of the paraumbilical incision adjacent to the umbilicus. Dense, scar-like fibrotic tissue was encountered, within which a neurovascular bundle was identified (arrow). (C) Intraoperative view of the right inguinal incision. No distinct nerve structure was identified directly beneath the incision at this level. (D) Inferiorly extended view of the right inguinal lesion shown in (C). A neuroma-like lesion embedded in fibrotic tissue was identified (arrow).

On initial examination, she had a clear, localized tender point to the right and slightly inferior to the umbilicus, and the Carnett sign was positive. We diagnosed ACNES and planned surgical neurectomy because her parents requested early surgical intervention. On the day before surgery, she newly complained of localized pain in the right groin at a brownish pigmented skin lesion. Based on these findings, we considered the possibility of an additional lesion involving a different nerve branch and performed neurectomy at two sites (Figure 1A).

**Figure 2:**
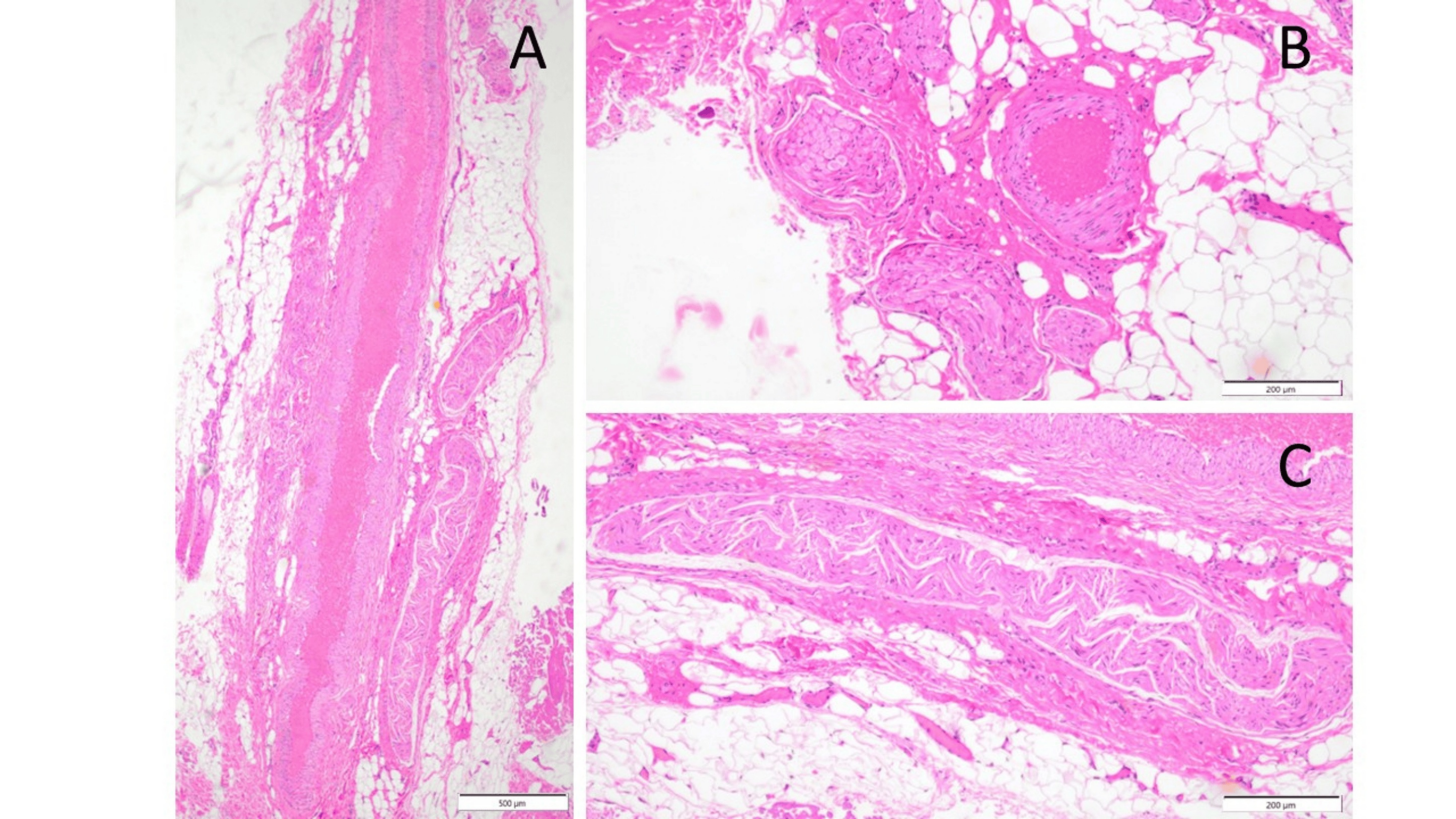
Histopathological findings of the paraumbilical lesion (hematoxylin and eosin staining) (A) Low-power longitudinal section of the paraumbilical lesion. A neurovascular bundle is observed, with the artery on the left and the nerve on the right, both closely apposed and surrounded by dense, collagen-rich fibrotic tissue, consistent with chronic perineural fibrosis (scale bar=500 μm). (B) Transverse section of the same lesion at higher magnification. The nerve and adjacent vessel are in close proximity, separated only by intervening collagen fibers, indicating intimate contact within the fibrotic stroma (scale bar=200 μm). (C) Higher-magnification view of the proximal portion of the nerve fascicle shown in (A). The nerve shows thickening and distortion at its root, with surrounding collagen deposition and no prominent inflammatory cell infiltration, supporting a non-inflammatory fibrotic process (scale bar=200 μm).

At the periumbilical site, a cutaneous nerve was identified immediately beneath the skin incision (Figure 1B). The nerve, including the fascia, was excised with scissors. Pathology demonstrated fibrosis surrounding the neurovascular bundle, thickening of the nerve fascicles, and close apposition of the nerve fascicles to an artery (Figure 2).

**Figure 3:**
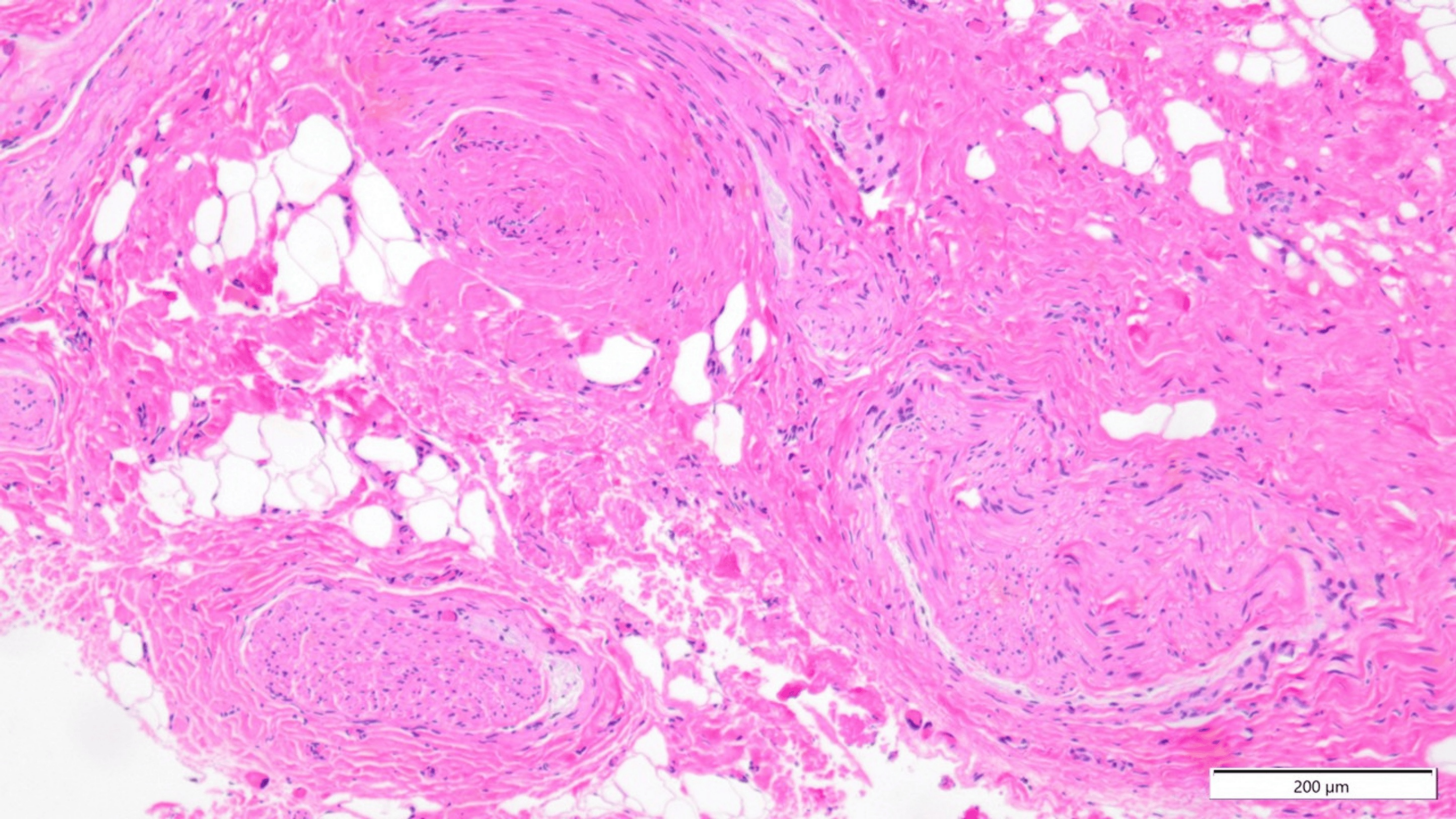
Histopathological findings of the right inguinal lesion (hematoxylin and eosin staining) Representative histological view showing multiple enlarged and irregularly arranged nerve fascicles embedded within dense, collagen-rich fibrotic tissue. The nerve fascicles are separated and distorted by intervening collagen fibers, consistent with traumatic neuroma-like changes associated with chronic fibrotic remodeling. No prominent inflammatory cell infiltration is observed (scale bar=200 μm).

At the right groin site, no cutaneous nerve was identified directly beneath the skin (Figure 1C). The subcutaneous tissue, together with the overlying pigmented skin lesion, was excised. Upon further exploration, a cutaneous nerve branch was identified arising from a point approximately 2 cm away from the pigmented lesion (a location considered to correspond to the LPEC needle insertion site) and extending cranially from the caudal side (Figure 1D). The nerve was gently tractioned and excised together with the fascia using scissors. Pathology of the subcutaneous tissue beneath the pigmented lesion demonstrated scar formation within adipose tissue and neuroma formation around the fascia. The neuroma consisted of whorled nerve fascicles intertwined with collagen fibers, forming a disorganized mass (Figure 3).

Postoperatively, pain at both sites resolved rapidly, and no recurrence was observed during the six-month follow-up period.

## Discussion

Chronic postoperative pain is a common and clinically relevant problem, and a subset of patients develop severe, localized neuropathic pain even when standard laboratory tests and imaging are unrevealing. A case report by Møller and Venzo described persistent postoperative scar pain caused by a cutaneous nerve embedded in scar tissue and emphasized that selective neurectomy can provide immediate symptom relief when a focal abdominal wall pain generator is correctly identified [8].

Our patient is noteworthy because two distinct abdominal wall pain generators appeared to coexist in the same child after bilateral LPEC: (1) a periumbilical lesion in which a neurovascular bundle was compressed within scar-like tissue (consistent with entrapment around a vessel within fibrotic tissue) and (2) a groin lesion with subcutaneous fat scarring and neuroma formation, anatomically corresponding to the presumed needle puncture tract used for LPEC, suggesting traumatic neuropathy. Importantly, postoperative abdominal wall neuropathic pain does not necessarily occur directly beneath a visible scar. For clinical vigilance, surgeons and clinicians should be aware that it may arise not only at the incision itself but also within the surrounding area (e.g., within approximately 2 cm), where local tissue remodeling and tethering can alter the course or mobility of adjacent cutaneous nerve branches and create a focal pain generator.

In our patient, the periumbilical pain site was not clearly aligned with the prior LPEC skin incisions. We therefore speculate that this lesion may have evolved independently of the surgical wound itself. The patient had a history of asthma, and perioperative wound pain and asthma-related symptoms (e.g., cough) may have contributed to chronically increased intra-abdominal pressure and repetitive abdominal wall muscle contraction. Such sustained mechanical stress could progressively compress vulnerable cutaneous nerve branches, leading to non-inflammatory perineurial/endoneurial fibrosis and microvascular compromise.

Histopathology in ACNES has not been fully characterized, and diverse findings have been reported [6]. Based on our current case (perineural fibrosis around a neurovascular bundle and thickened nerve fascicles) and prior observations, although derived from limited evidence, we propose that non-inflammatory, progressive fibrosis may represent a fundamental underlying substrate of ACNES. Within this framework, traumatic neuroma may share certain fibrotic characteristics with entrapment-related lesions, although they are considered distinct pathological processes. Because perineural fibrosis is largely collagen-based, it consists predominantly of collagen, a protein known to exhibit piezoelectric properties, generating electrical potentials in response to mechanical stress [9]. This raises the possibility that mechanically stressed fibrotic tissue may not be electrically inert. We therefore propose a speculative hypothesis that disruption of the perineurium together with collagen-rich fibrosis may create a potential electromechanical microenvironment in which motion- or pressure-related triggers (e.g., body movement or arterial pulsation) induce transient electrical activity that manifests clinically as sudden "electric shock-like" pain. This concept remains theoretical and has not been directly demonstrated in abdominal wall nerves and warrants further investigation. However, similar electromechanical mechanisms might also be relevant to other pain disorders, such as trigeminal neuralgia, migraine, and other forms of chronic pain [10-12].

Experimental evidence supports a link between peripheral nerve dysfunction, vascular abnormalities, and persistent neuropathic pain. In a traumatic peripheral nerve injury model, Lim et al. reported persistent microvascular dysfunction and endoneurial hypoxia accompanied by fibrosis, changes that were associated with neuronal hyperexcitability [13]. We propose that an analogous vicious cycle may occur in abdominal wall nerves in susceptible patients: repeated mechanical compression may impair microvascular flow, promote fibrosis, and ultimately produce an irreversible state beyond a certain point, at which sudden paroxysmal "electric shock-like" pain emerges. In our case, pathology and intraoperative findings suggested close apposition of the nerve fascicle and accompanying vessel at the periumbilical site. Once the fascicle is injured and mechanically constrained, direct exposure to arterial pulsation may further aggravate ectopic firing, potentially explaining the abrupt lancinating pain. Clinically, the patient experienced sudden electric shock-like pain severe enough to prevent school attendance, highlighting the functional burden even in pediatric cases.

The report by Møller and Venzo also provides a practical framework for differential diagnosis when postoperative pain is sharply localized to the abdominal wall [8]. In clinical practice, ACNES is suggested by a unilateral, well-circumscribed tender spot (typically near the lateral border of the rectus muscle), a positive Carnett sign (increased pain with abdominal wall tensing), and sometimes a positive Tinel sign at the point of maximal tenderness. Temporary pain relief after local anesthetic injection at the tender spot is another useful confirmatory test. In our case, the prominent focal tenderness and positive Carnett sign at the periumbilical site strongly supported an abdominal wall origin and helped avoid further visceral-focused investigations. Traumatic neuroma is an important postoperative mimic of ACNES with a similar clinical presentation [14]. It may develop after nerve injury, particularly when a damaged nerve is exposed to a healing wound and becomes incorporated into fibrotic tissue, and it can be promoted by traction or repeated irritation. In the setting of LPEC, needle puncture and suture passage create potential sites for minor peripheral nerve trauma along the puncture tract; therefore, neuroma-related pain should be considered when symptoms localize to a port, puncture, or suture pathway.

From a surgical perspective, selective neurectomy remains a reasonable option for patients with persistent, function-limiting focal abdominal wall pain refractory to conservative measures [15]. In addition to resection, relocation of the proximal nerve end away from the scar field (e.g., burying it into muscle) has been advocated to reduce the risk of re-entrapment and recurrent neuroma-related pain [16]. Our patient experienced prompt and sustained relief after neurectomy at both pain sites, supporting a lesion-directed approach.

Finally, this case underlines that postoperative abdominal wall pain should not be assumed to be a single-lesion phenomenon. When the history or examination suggests more than one focal pain point, clinicians should evaluate each site systematically, as multiple lesions with different mechanisms may coexist.

## Conclusions

This case highlights that distinct mechanisms of postoperative abdominal wall neuropathic pain may coexist in a single patient after LPEC. Non-inflammatory fibrotic entrapment of a cutaneous nerve and traumatic neuroma formation along a puncture tract can present with similar focal pain symptoms but arise from different pathological processes. Importantly, postoperative abdominal wall pain does not necessarily localize directly beneath visible scars, and careful physical examination is essential to identify focal pain generators. Recognition of these distinct mechanisms allows lesion-directed surgical management, such as selective neurectomy, which can result in prompt and sustained symptom relief. Surgeons and clinicians should remain vigilant for multiple potential pain sources when evaluating persistent, localized abdominal wall pain following minimally invasive procedures.
